# Serum Uric Acid and Mortality Risk in Chronic Kidney Disease: A Dose–Response Analysis

**DOI:** 10.3390/jcm15145479

**Published:** 2026-07-13

**Authors:** Rasha Babiker, Hassan Khammas, Nada Tawfig Hashim, Imran Rangraze, Amin S. I. Banaga, Dania Elgasim, Reem Ismail Nooh, Asmaa A. Muhammed, Ashfaq Ahmad Shah Bukhari, B. K. Manjunatha Goud, Mohamed El-Tanani, Tarig H. Merghani, Mohammed Naveed

**Affiliations:** 1 Department of Physiology, RAK College of Medical Sciences, Ras Al Khaimah Medical and Health Sciences University, Ras Al Khaimah P.O. Box 11172, United Arab Emirates; asmaa@rakmhsu.ac.ae (A.A.M.); ashfaq.ahmad@rakmhsu.ac.ae (A.A.S.B.); tarig@rakmhsu.ac.ae (T.H.M.); 2 Sheikh Khalifa Hospital, Fujairah P.O. Box 3344, United Arab Emirates; hassan.khammas@ehs.gov.ae; 3 Department of Periodontics, RAK College of Dental Sciences, RAK Medical & Health Sciences University, Ras Al Khaimah 12973, United Arab Emirates; nada.tawfig@rakmhsu.ac.ae; 4 Department of Internal Medicine, RAK College of Medical Sciences, Ras Al Khaimah Medical and Health Sciences University, Ras Al Khaimah P.O. Box 11172, United Arab Emirates; imranrashid@rakmhsu.ac.ae; 5 Faculty of Medicine, National University, Khartoum 11115, Sudan; amin.banaga@gmail.com; 6 RAK College of Medical Sciences, Ras Al Khaimah Medical and Health Sciences University, Ras Al Khaimah P.O. Box 11172, United Arab Emirates; daniaelgasim@hotmail.com (D.E.); reemxnooh@gmail.com (R.I.N.); 7 Department of Biochemistry, RAK College of Medical Sciences, Ras Al Khaimah Medical and Health Sciences University, Ras Al Khaimah 11172, United Arab Emirates; manjunatha@rakmhsu.ac.ae; 8 RAK College of Pharmacy, RAK Medical and Health Sciences University, Ras Al Khaimah 11172, United Arab Emirates; eltanani@rakmhsu.ac.ae; 9 Department of Paediatrics, Fujairah Hospital, Emirates Health Services, Fujairah P.O. Box 10, United Arab Emirates; mohammed.naveed@ehs.gov.ae

**Keywords:** hyperuricemia, chronic kidney disease, cardiovascular disease, uric acid, cardiorenal syndrome

## Abstract

**Background/Objectives**: Hyperuricemia is highly prevalent in chronic kidney disease (CKD). It has been linked to increased cardiovascular and mortality risk. However, its independent prognostic significance and dose–response relationship with adverse outcomes remain incompletely understood. **Methods**: This retrospective cohort study included 794 patients with CKD. Hyperuricemia was defined using sex-specific thresholds. The association between hyperuricemia, cardiac events and mortality was tested using a multivariable logistic regression test. Serum uric acid was analyzed as both a categorical and a continuous variable to assess dose–response relationships. **Results**: Hyperuricemia was present in 44.8% of patients. In multivariable analysis, hyperuricemia was associated with increased odds of cardiac events, although this did not reach statistical significance (OR 1.49, 95% CI 0.98–2.27, *p* = 0.061). However, hyperuricemia independently predicted mortality (OR 1.98, 95% CI 1.04–3.78, *p* = 0.039), and importantly, a dose–response relationship was observed between serum uric acid and adverse outcomes. Each 100 μmol/L increase in serum uric acid was associated with a 22% increase in cardiac event risk (OR 1.22, *p* = 0.011) and a 29% increase in mortality risk (OR 1.29, *p* = 0.004). Quartile analysis revealed that patients in the highest uric acid quartile had more than three-fold higher mortality compared with the lowest quartile (OR 3.13, 95% CI 1.26–7.79, *p* = 0.014). **Conclusions**: Hyperuricemia independently predicts mortality and also demonstrates a significant dose–response relationship with adverse outcomes in CKD. The results support serum uric acid as a clinically meaningful biomarker for mortality risk grading in CKD populations.

## 1. Introduction

Chronic kidney disease (CKD) is a major global health challenge, affecting approximately 10–15% of the adult population and contributes substantially to cardiovascular morbidity and premature mortality [1]. Patients with CKD experience markedly elevated cardiovascular (CVD) risk compared with the general population, and cardiovascular disease accounts for nearly half of all deaths in this population [2]. This high risk arises from a complex interplay between common, well-known cardiovascular risk factors, including hypertension and diabetes mellitus, and CKD-recent pathophysiological mechanisms such as endothelial dysfunction, oxidative stress, inflammation, vascular calcification, and neurohormonal activation [3,4]. Despite advances in cardiovascular risk management, CKD patients continue to experience poor outcomes. These poor outcomes underscore the need to identify additional biomarkers that can improve risk levels and provide insight into underlying disease mechanisms [5]. Current cardiovascular risk-stratification approaches in CKD rely predominantly on established variables such as age, estimated glomerular filtration rate, and proteinuria, yet these do not fully account for the residual risk observed in this population; whether serum uric acid—an inexpensive and routinely measured analyte—adds prognostic information beyond these variables remains uncertain and is the focus of the present study.

**Figure 1:**
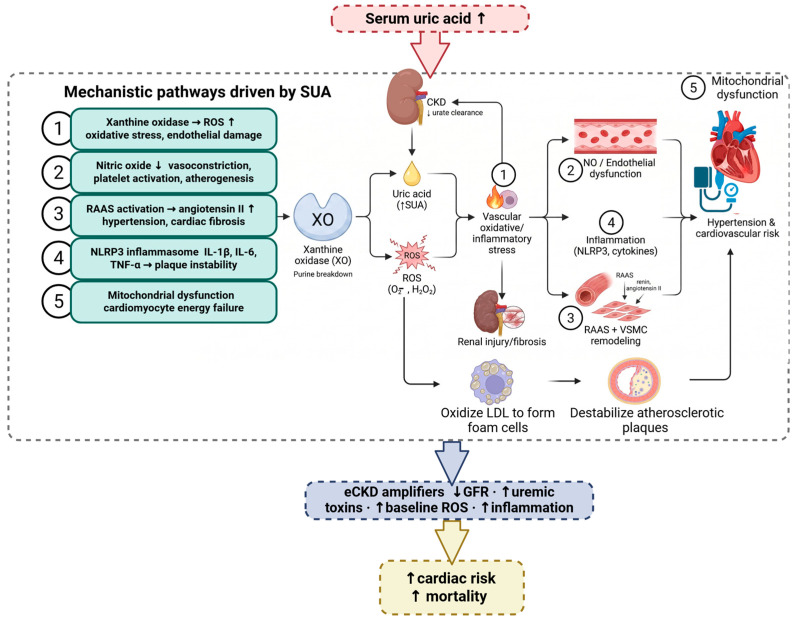
This figure explains the suggested mechanistic pathways implicating high serum uric acid (SUA) in cardiovascular and renal complications in CKD. Serum uric acid is often elevated in CKD partly due to decreased renal urate clearance, and this promotes vascular oxidative and inflammatory stress through several inter-related mechanisms. (1) XO produces uric acid, which can enhance the production of ROS and thus causes oxidative stress and endothelial injury. (2) Decreased nitric oxide availability leads to endothelium dysfunction, vasoconstriction, platelet activation and disturbance of vascular homeostasis. (3) Activation of the renin–angiotensin–aldosterone system leads to stimulation of angiotensin II signaling, which causes VSMC remodeling, hypertension, and cardiac fibrosis. (4) Uric acid induces NLRP3 inflammasome activation, which promotes the secretion of pro-inflammatory cytokines that promote vascular inflammation and plaque instability, such as IL-1β, IL-6, and TNF-α. (5) Mitochondrial dysfunction disrupts cellular energy metabolism and aggravates cardiovascular injury. This results in renal injury and fibrosis, low-density-lipoprotein particle oxidation, foam cell formation, destabilization of atherosclerotic plaques and increased cardiovascular risk. In advanced CKD, reduction in glomerular filtration rate, accumulation of uremic toxins, increased baseline production of ROS, and persistent inflammation contribute to the exacerbation of these pathological pathways, further increasing cardiovascular morbidity and mortality. Abbreviations: SUA, serum uric acid; CKD, chronic kidney disease; XO, xanthine oxidase; ROS, reactive oxygen species; NO, nitric oxide; RAAS, renin–angiotensin–aldosterone system; VSMC, vascular smooth muscle cell; NLRP3, NOD-like receptor family pyrin domain-containing 3; LDL, low-density lipoprotein; GFR, glomerular filtration rate; IL, interleukin; TNF-α, tumor necrosis factor-alpha.

Serum uric acid (SUA) has emerged as a potentially important biomarker in cardiorenal disease. Hyperuricemia is highly prevalent in CKD due to impaired renal excretion and altered purine metabolism. As a result of this impairment and progressive accumulation of uric acid, renal function declines [6]. Uric acid has been implicated in multiple pathophysiological processes relevant to cardiovascular injury and systemic dysfunction [7]. Experimental studies have demonstrated that nitric oxide bioavailability decreases due to the elevated uric acid, which promotes endothelial dysfunction, increases oxidative stress through activation of NADPH oxidase, and stimulates inflammatory signaling pathways, such as interleukin-1β and tumor necrosis factor-α [8,9]. Uric acid also activates the RAAS system (Figure 1).

Various mechanisms have been suggested in describing how the RAAS system contributes to hyperuricemia, such as vasoconstriction, hypertension, vascular remodeling, and progression of renal disease. These mechanisms have been proposed as pathways by which hyperuricemia could be associated with adverse cardiovascular and systemic outcomes, rather than functioning solely as a marker of reduced kidney function [10,11] (Figure 1).

Beyond its role as a product of impaired renal urate handling, serum uric acid is increasingly recognized as a component of a broader cardiometabolic phenotype. Elevated concentrations frequently co-occur with metabolic syndrome, adverse cardiometabolic profiles, obesity-related phenotypes, and alterations in body composition, and uric acid has been identified as a discriminating biomarker of metabolically unhealthy status independent of adiposity [12,13]. Whether serum uric acid represents an independent contributor to adverse cardiovascular and mortality outcomes or is primarily a marker of underlying disease severity and metabolic dysregulation remains unresolved and actively debated, with the current literature providing conflicting evidence. Within CKD specifically, the extent to which serum uric acid carries prognostic information beyond categorical definitions of hyperuricemia and whether any such association follows a graded dose–response pattern have not been fully characterized. The present study was therefore designed to address this gap by evaluating the independent association of serum uric acid with cardiovascular events and all-cause mortality in a CKD cohort, analyzing uric acid both categorically and as a continuous, quartile-based measure to test for a dose–response relationship. In doing so, this study is intended to add real-world, confirmatory evidence from a single-center cohort in the United Arab Emirates—a Middle Eastern population under-represented in the existing literature—rather than to propose a new mechanistic theory or prediction model.

## 2. Materials and Methods

### 2.1. Study Design and Population

**Figure 2:**
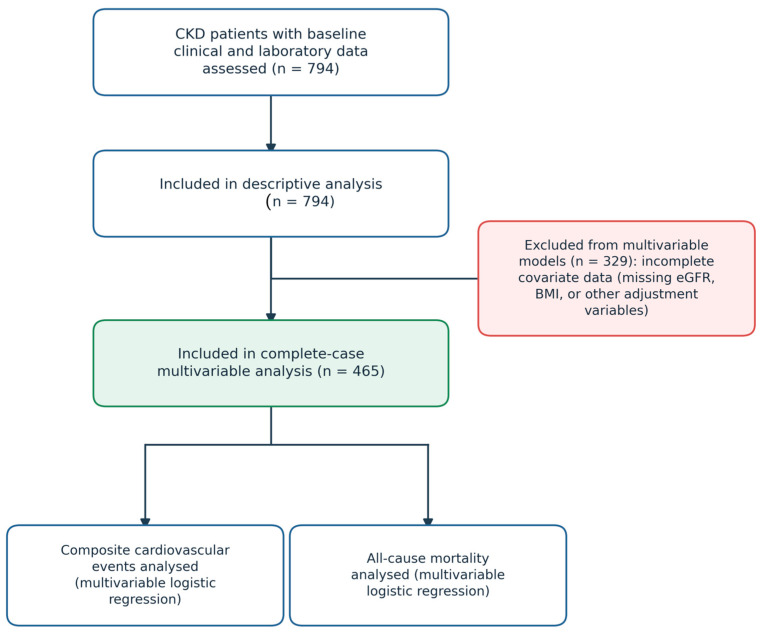
Study participant flow diagram (STROBE). Of 794 patients with CKD assessed, 329 with incomplete covariate data were excluded from the multivariable models, leaving 465 in the complete-case analysis.

This retrospective observational cohort study was conducted at the Fujairah Nephrology and Dialysis Department, United Arab Emirates. This study included adult patients diagnosed with chronic kidney disease (CKD) who attended their first nephrology visit between January 2018 and December 2021, the period defining the observation window over which cardiovascular events and mortality were ascertained. A total of 794 patients with baseline clinical and laboratory data were included in the descriptive analysis (Figure 2). Chronic kidney disease was defined according to the Kidney Disease: Improving Global Outcomes (KDIGO) criteria, namely, an estimated glomerular filtration rate below 60 mL/min/1.73 m^2^ and/or markers of kidney damage persisting for at least three months. In the present cohort, among the 675 patients with an available eGFR, the distribution across KDIGO categories was G1 2.1%, G2 8.3%, G3a 11.1%, G3b 17.5%, G4 24.7%, and G5 36.3%, confirming a predominantly advanced-CKD population (approximately 61% in categories G4–G5). Maintenance dialysis or end-stage renal disease was documented in 35 patients (4.4%) and kidney transplantation in 9 (1.1%); as these were captured from free-text records, they may be under-ascertained. Exploratory patterns across eGFR categories and by dialysis status were consistent with the overall findings.

Adult patients of both sexes who were eligible to participate were included in the study. Patients were included based on the accessibility of SUA levels, renal function and cardiovascular data at the time of initial evaluation. Patients with incomplete essential baseline data were excluded from multivariable analyses but were retained in descriptive analyses where applicable.

Ethical approval was obtained from the Ministry of Health and Prevention Research Ethics Committee (MOHAP/DXB REC/D.D-J/No. 138/2022) and the Research Ethics Committee of Ras Al Khaimah Medical and Health Sciences University. All procedures were conducted in accordance to the ethical standards of the institutional and national levels. Patient confidentiality was strictly maintained, and all data were anonymized prior to analysis.

### 2.2. Data Collection and Management

All clinical data were collected retrospectively from the hospital’s electronic medical record (EMR) system. Patient identifiers were removed to preserve confidentiality of patients, and each record was assigned a unique anonymous study identification number.

At the first nephrology visit, the baseline variables collected included demographic variables (age and sex), comorbidities (hypertension and diabetes mellitus), body mass index (BMI), estimated glomerular filtration rate (eGFR) and SUA levels. All analyses used these baseline measures as index scores.

Cardiovascular data were obtained by systematic review of clinical records, including documented diagnoses, symptoms, and diagnostic findings obtained during routine clinical evaluation. Cardiovascular data included documented diagnoses of angina pectoris, congestive heart failure, myocardial infarction, coronary artery disease, arrhythmia, cardiomyopathy, stroke, and abnormal electrocardiographic findings, as well as documented cardiovascular symptoms and cardiology consultations. Mortality status was determined from clinical records of death during the observation period.

### 2.3. Definition of Hyperuricemia

Hyperuricemia was defined by sex-specific serum uric acid cut-offs which fit with established clinical criteria. Hyperuricemia was defined as SUA > 420 μmol/L for men and >360 μmol/L for women. This classification is often used in epidemiological and clinical studies, indicating physiological differences in uric acid metabolism between sexes [14,15,16].

To evaluate dose–response relationships, serum uric acid was also analyzed as a continuous variable and categorized into quartiles based on its distribution within the study population.

### 2.4. Outcomes

Two primary outcomes were a composite cardiovascular event indicator, defined based on documented cardiovascular diagnoses, clinical findings, and diagnostic investigations. Major cardiovascular events included angina pectoris, myocardial infarction, arrhythmia, stroke, heart failure, coronary artery disease, and cardiomyopathy. Secondary cardiac indicators included abnormal electrocardiographic findings and clinically documented cardiac symptoms. The composite outcome was constructed to reflect overall cardiovascular burden. This composite was adopted because, in a retrospective single-center dataset assembled from routine electronic records, individual major events were too infrequent to model reliably in isolation, and combining them provided adequate statistical power to examine associations with serum uric acid. To limit the possibility that clinically milder components (abnormal electrocardiographic findings and documented cardiac symptoms) would obscure associations with hard events, the major cardiovascular events listed above were also examined separately where the available data permitted; the heterogeneity of the composite endpoint is nonetheless acknowledged as a limitation.

The secondary outcome was all-cause mortality, defined as documented death recorded in the clinical medical record system.

These outcomes were analyzed separately to evaluate the independent association between SUA levels and cardiovascular and mortality risk.

### 2.5. Statistical Analysis

Statistical analyses were tested by using IBM SPSS Statistics version 30.0 (IBM Corp., Armonk, NY, USA). Continuous variables were tested for normality and expressed as means ± standard deviation (SD). The categorical variables are reported as percentages and frequencies.

Multivariable logistic regression analyses were performed to assess the independent association between hyperuricemia and study outcomes.

The models were adjusted for clinically relevant covariates, such as age, sex, estimated glomerular filtration rate (eGFR), diabetes mellitus, hypertension, and body mass index. Results are presented as odds ratios (ORs) with 95% confidence intervals (95% CIs). Multivariable modeling was performed using complete-case analysis. Complete-case analysis was used because covariates required for adjustment (notably body mass index and estimated glomerular filtration rate) were missing for a proportion of patients, reducing the sample available for multivariable modeling from 794 to 465; patients with incomplete covariate data were excluded only from the adjusted models and were retained in the descriptive analysis. Multiple imputation was considered as an alternative; however, because missingness arose predominantly from variables that were not recorded at the index visit rather than from data missing at random, imputation was not applied in the primary analysis, and the potential for selection bias introduced by the complete-case approach is acknowledged as a limitation. Outcomes were modeled using multivariable logistic regression rather than Cox proportional-hazards regression because reliable individual-level dates of events and censoring could not be retrieved from the retrospective records, which precluded the calculation of person-time and the valid fitting of time-to-event models; events were therefore analyzed as binary outcomes ascertained over the observation window. Model discrimination was interpreted conservatively, and the modest discrimination obtained for the cardiovascular composite in particular is not presented as evidence of clinical prediction utility. Covariates were pre-specified on clinical grounds (age, sex, eGFR, diabetes mellitus, hypertension, and body mass index) rather than selected by data-driven procedures. Because the mortality model included several covariates relative to a limited number of deaths, the events-per-variable ratio was low, and the possibility of model overfitting is acknowledged; formal assessment of multicollinearity and of interaction terms was not undertaken. The cohort included 66 deaths (728 survivors) and 414 composite cardiovascular events (380 without an event); the events and non-events entering each complete-case model are constrained by the covariate completeness described above.

To assess dose–response relationships, SUA was additionally analyzed as a continuous variable, with odds ratios calculated per 100 μmol/L increase. Quartile analysis was also performed by categorizing serum uric acid into four quartiles based on the study population distribution, using the lowest quartile as the reference group.

Model discrimination was analyzed with ROC curve analysis, and we measured predictive performance with the area under the curve (AUC) and associated confidence intervals. Optimal probability thresholds were used to calculate sensitivity, specificity, and classification accuracy. Calibration was assessed using calibration plots comparing observed and predicted probabilities of events.

Statistical tests were two-sided, and a *p*-value < 0.05 was considered statistically significant.

## 3. Results

### 3.1. Study Population Characteristics

A total of 794 CKD patients were included in the final analysis. The mean age was 68.5 ± 16.8 years. Males comprised 60.5%, and females were 39.5% of the cohort. Hyperuricemia was present in 44.8% of patients. Hypertension and diabetes mellitus were highly prevalent, affecting 67.4% and 53.0% of participants, respectively. Mean SUA concentration was 401.3 ± 162.5 μmol/L, and mean estimated glomerular filtration rate (eGFR) was 28.7 ± 22.7 mL/min/1.73 m^2^. The eGFR was consistent with moderate-to-severe renal impairment.

Cardiac events were documented in 52.1% of patients, reflecting the substantial cardiovascular burden in this CKD population. Overall mortality was 8.3%. Of the 794 patients, 465 (58.6%) had complete data for all adjustment covariates and were included in the multivariable models, whereas 329 (41.4%) had one or more missing covariates and were included only in the descriptive analysis (Figure 2). Baseline characteristics stratified by hyperuricemia status and by survival status are presented in Supplementary Tables S1 and S2. The most frequently missing covariates were eGFR (120 patients, 15.1%), body mass index (80, 10.1%), lipid measures (71–85, approximately 9–11%), and C-reactive protein (43, 5.4%), whereas serum uric acid was missing in only five patients (0.6%). Smoking was documented in 66 patients (8.3%); mean lipid concentrations were total cholesterol, 3.7 ± 1.3; LDL, 2.0 ± 1.0; HDL, 1.2 ± 0.4; and triglycerides, 1.3 ± 0.8 mmol/L. And median C-reactive protein was 13.7 mg/L (IQR 3.0–56.2). These variables were used in the stratified comparisons (Tables S1 and S2) and the sensitivity analysis (Table 1).

### 3.2. Hyperuricemia and Cardiac Events

The complete-case-data model (*N* = 465), adjusted for age, sex, eGFR, diabetes mellitus, hypertension, and body mass index, was analyzed by multivariable logistic regression analysis. Hyperuricemia was associated with increased odds of composite cardiac events; however, this association did not reach statistical significance after adjustment (OR 1.49, 95% CI 0.98–2.27, *p* = 0.061).

Male sex was a strong independent predictor, with males having more than twice the odds of cardiac events compared with females (OR 2.16, *p* < 0.001). Age was independently associated with cardiac events, with each additional year increasing risk (OR 1.02, *p* = 0.0008). Hypertension was associated with an increased risk of cardiac events (OR 2.87, *p* = 0.001) independently. However, in the adjusted model, eGFR, diabetes mellitus, and body mass index were not independently related to cardiac events (Table 2).

### 3.3. Hyperuricemia and Death

Hyperuricemia was associated with an increased risk of death (OR 1.98, 95% CI 1.04 to 3.78, *p* = 0.039) independently. Mortality was significantly associated with age and borderline-associated with eGFR.

Importantly, we found a clear dose–response relationship between serum uric acid and risk of death. An increase in serum uric acid of 100 μmol/L was associated with a 29% increase in risk of mortality after adjustment for confounders (Table 3).

### 3.4. Dose–Response Relationship Between SUA and Outcomes

A significant dose–response relationship between SUA and adverse outcomes was revealed by continuous analysis. In the adjusted models, each 100 μmol/L increase in serum uric acid was associated with a 22% increase in the odds of cardiac events (OR 1.22, 95% CI 1.05–1.42, *p* = 0.011) and a 29% increase in the odds of mortality (OR 1.29, 95% CI 1.09–1.53, *p* = 0.004). Whereas categorically defined hyperuricemia was not independently associated with cardiac events after adjustment (*p* = 0.061), serum uric acid modeled as a continuous variable was significantly associated with both outcomes, indicating that the graded relationship is not fully captured by binary thresholds.

Quartile analysis further confirmed this relationship, with patients in the highest uric acid quartile exhibiting more than three-fold higher mortality compared with those in the lowest quartile (Table 4).

### 3.5. Quartile Analysis of Serum Uric Acid and Mortality

**Figure 3:**
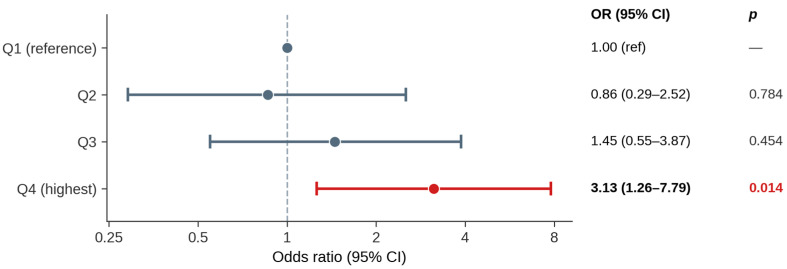
Odds ratios (ORs) and 95% confidence intervals (CIs) were estimated using Q1 as the reference category. Compared with Q1, participants in Q2 (OR = 0.86, 95% CI: 0.29–2.52, *p* = 0.784) and Q3 (OR = 1.45, 95% CI: 0.55–3.87, *p* = 0.454) showed no significant increase in risk. In contrast, individuals in the highest quartile (Q4) had a significantly greater risk of the outcome (OR = 3.13, 95% CI: 1.26–7.79, *p* = 0.014). The dashed vertical line indicates the null value (OR = 1.0), with error bars representing 95% confidence intervals. Significant risk is shown in bold and red color.

The quartile analysis demonstrated the presence of a dose–response relationship between SUA and mortality risk. Patients in the highest quartile exhibited more than three-fold higher adjusted odds of death compared with those in the lowest quartile (OR 3.13, 95% CI 1.26–7.79, *p* = 0.014). The intermediate quartiles (Q2 and Q3) were not significantly associated with mortality relative to the lowest quartile. For reproducibility, the serum uric acid ranges defining the quartiles in the study population were: Q1, ≤294 µmol/L; Q2, 295–382 µmol/L; Q3, 383–484 µmol/L; and Q4, ≥485 µmol/L. Restricted cubic spline modeling is recommended as a complementary approach in future analyses to formally evaluate potential non-linearity (Figure 3). In a sensitivity analysis additionally adjusting for urate-lowering therapy, diuretic use, and smoking status (available for 636 patients with complete data on these covariates), the association between hyperuricemia and mortality remained significant (adjusted OR 1.96, 95% CI 1.02–3.75), as did the continuous per-100 µmol/L association (OR 1.30, 95% CI 1.09–1.54). Urate-lowering therapy (allopurinol) was recorded in 46 patients and diuretics in 262, and neither materially attenuated the serum uric acid–mortality association, indicating robustness to medication confounding.

### 3.6. Model Discrimination and Classification Performance

The area under the curve was 0.630 (95% CI 0.576–0.697), suggesting moderate discriminative ability of the multivariable model for cardiac events. The model at the optimal probability cut-off (0.113) had a sensitivity of 65.1%, a specificity of 54.6% and an overall classification accuracy of 55.9%.

The multivariable model demonstrated good discriminative performance for mortality prediction, with an AUC of 0.748 (95% CI 0.680–0.805), indicating substantially stronger predictive capability.

**Figure 4:**
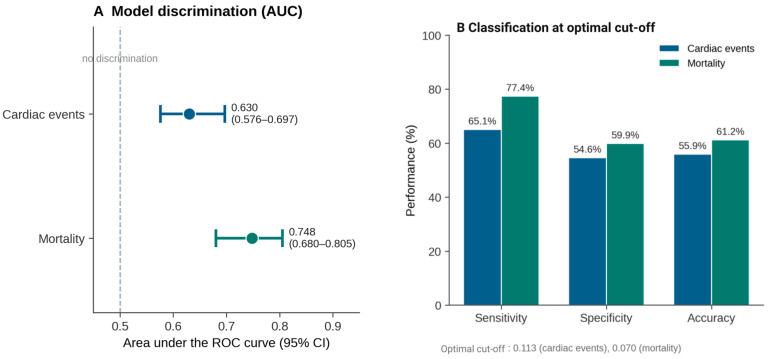
Predictive performance of the model for cardiac events and mortality. (**A**) Presents the area under the receiver operating characteristic curve (AUC) point estimates with 95% confidence intervals for each outcome rather than the full ROC curves. The model demonstrated moderate discrimination for cardiac events (AUC = 0.630, 95% CI: 0.576–0.697) and good discrimination for mortality (AUC = 0.748, 95% CI: 0.680–0.805). (**B**) Classification performance at the optimal cut-off values (0.113 for cardiac events and 0.070 for mortality). Sensitivity, specificity, and overall accuracy are shown for each outcome. The model achieved higher sensitivity, specificity, and accuracy for mortality prediction compared with cardiac event prediction.

At the optimal cut-off probability (0.070), sensitivity was 77.4%, specificity was 59.9%, and overall classification accuracy was 61.2% (Figure 4).

Calibration analysis showed good consistency between predicted and observed mortality risk, supporting the reliability of the model.

## 4. Discussion

In this cohort study, we demonstrated that hyperuricemia independently predicts mortality. On the other hand, hyperuricemia was not independently associated with composite cardiac events [17,18], when analyzed as a binary variable after adjustment for demographic and clinical confounders. However, continuous and quartile-based analyses revealed a clear dose–response relationship between SUA levels and adverse outcomes. Patients in the high-SUA quartile showed more than three-fold higher mortality compared with the lowest quartile. This indicates that each incremental increase in serum uric acid was associated with progressively higher mortality risk. These findings suggest that serum uric acid functions not merely as a categorical risk factor but as a graded biomarker of systemic vulnerability and adverse prognosis in CKD. Our findings regarding mortality are consistent with a substantial body of epidemiological evidence demonstrating an independent association between elevated serum uric acid and all-cause mortality in CKD and general populations. Studies on NHANES and CKD-specific registries have shown that hyperuricemia is associated with increased mortality risk even after adjustment for renal function and traditional cardiovascular risk factors [19,20,21].

The mortality model showed acceptable discrimination (AUC 0.748), whereas discrimination for the cardiovascular composite was modest (AUC 0.630). These values indicate that serum uric acid and related covariates carry prognostic information for mortality, but they do not by themselves establish clinical prediction utility, and the discriminative performance—particularly for cardiovascular events—should not be overstated. Accordingly, these findings should be regarded as hypothesis-generating associations rather than as evidence that serum uric acid is an independent, clinically actionable risk factor, particularly for cardiovascular events, where the adjusted association was non-significant and discrimination was weak. This finding suggests that serum uric acid provides meaningful prognostic information and may add to the identification of high-risk patients in addition to conventional clinical variables.

A finding reported by the Chronic Renal Insufficiency Cohort (CRIC) Study is that elevated SUA levels were independently associated with increased mortality risk, supporting the concept that hyperuricemia reflects systemic metabolic and inflammatory dysregulation rather than reduced renal excretion [10]. Our observation of a strong dose–response relationship further strengthens this association, as graded increases in serum uric acid corresponded to progressively higher mortality risk; this pattern aligns with several prior studies that have reported attenuation of cardiovascular risk associations after controlling for renal function and other confounders [22,23]. This pattern supports biological plausibility and argues against the relationship is solely due to residual confounding.

Uric acid is involved in several pathophysiological pathways that contribute to adverse cardiovascular and systemic outcomes. Experimental studies indicate that elevated uric acid may impair endothelial function by reducing nitric oxide bioavailability, which is associated with impaired vascular relaxation and increased vascular stiffness.

Uric acid also induces oxidative stress by activation of NADPH oxidase, increasing the production of reactive oxygen species and mitochondrial dysfunction. These oxidative processes lead to endothelial injury, inflammation and vascular remodeling [24,25]. Uric acid has also been shown to activate the renin–angiotensin–aldosterone system (RAAS) which is important in the development of hypertension, vascular damage and progression of renal disease. RAAS activation induces vasoconstriction, sodium retention and fibrosis, which leads to progression of both cardiovascular and renal deterioration [7,26]. Impaired uric acid excretion, particularly in CKD, results in progressive accumulation and magnifies these maladaptive mechanisms.

Uric acid also promotes inflammatory signaling via activation of pro-inflammatory cytokines such as IL-6, IL-1β, and TNF-α, which all promote systemic inflammation, endothelial injury, and adverse cardiovascular remodeling [27].

Beyond the direct biological effects, hyperuricemia may be a marker of more generalized metabolic dysregulation and systemic disease severity. Elevated uric acid is strongly linked to insulin resistance, metabolic syndrome, oxidative stress and chronic inflammation, which are contributing factors to adverse clinical outcomes [28]. In CKD populations, hyperuricemia may thus serve as an integrative marker for cumulative physiologic stress and metabolic burden rather than a single isolated pathologic process. This is consistent with our finding that hyperuricemia was more strongly and consistently associated with mortality than with discrete cardiovascular events, suggesting that uric acid reflects general systemic vulnerability. More broadly, the association between serum uric acid and mortality is best interpreted in the context of complex metabolic dysregulation rather than impaired renal clearance alone. Elevated uric acid is closely linked to obesity-related metabolic abnormalities, insulin resistance, visceral adiposity, and hepatic dysfunction, and in analyses stratified by comparable obesity status, higher uric acid has been shown to independently distinguish metabolically unhealthy from metabolically healthy phenotypes, indicating that cardiometabolic risk is not explained by adiposity alone [13]. Inter-individual variation in uric acid also reflects genetic and hormonal influences: menopause-related hormonal changes and common variants in genes involved in estrogen signaling and in lipid and carbohydrate metabolism (for example, ESR1 and MLXIPL) have been associated with circulating uric acid concentrations [29]. Interpreting serum uric acid in CKD as an integrative marker of cumulative metabolic burden therefore cautions against assuming that its association with mortality reflects a single, uric acid–specific causal pathway.

Our findings are also consistent with emerging evidence that in advanced-CKD cohorts, the association of hyperuricemia with mortality may be more reliable than its association with incident cardiovascular events. This pattern may reflect competing-risk dynamics, given that patients with CKD are exposed to multiple overlapping insults, including chronic inflammation, anemia, disordered mineral metabolism and the accumulation of uremic toxins. In this context, uric acid could have a role in cumulative physiological decline and general mortality, rather than only through discrete cardiovascular events. Potential mechanisms include mitochondrial dysfunction, impaired cellular repair, and chronic inflammation, all of which may increase susceptibility to fatal consequences [30,31].

Our study provides particularly important clinical insights into the dose–response relationship. The observed graded association of risk of mortality with increasing levels of uric acid suggests that risk stratification with continuous uric acid measures may provide clinically meaningful prognostic information beyond categorical definitions of hyperuricemia. This finding is directly relevant to clinical risk prediction and underscores the potential value of including serum uric acid in prognostic models in CKD populations.

Our findings also add to the ongoing debate on whether hyperuricemia is a causal risk factor or a marker of disease severity. Experimental support exists for causal biological mechanisms, but clinical evidence is mixed. Our findings indicate that hyperuricemia is mechanistically linked to oxidative stress and vascular dysfunction, as well as a marker of underlying metabolic and renal impairment.

The strong dose–response relationship supports a biologically meaningful association and the attenuation of binary associations after adjustment suggests that uric acid is part of a wider network of pathophysiological processes [32].

Several limitations should be considered when interpreting these findings. First, reliable individual-level follow-up duration, event dates, and censoring information could not be retrieved from the retrospective records; consequently, time-to-event (survival) and competing-risk analyses were not feasible, outcomes were modeled as binary events over the observation window, and serum uric acid was measured at a single baseline time point. Second, the multivariable models were restricted to patients with complete covariate data (465 of 794), which may introduce selection bias and reduce statistical power; a formal comparison of included and excluded patients is warranted, and multiple imputation was not undertaken. Third, the composite cardiovascular endpoint combined heterogeneous outcomes ranging from major events to abnormal electrocardiographic findings and symptoms, which may dilute associations with hard events, and model discrimination for the cardiovascular composite was modest. Fourth, this study lacked information on several factors that may influence serum uric acid and outcomes, including dietary habits, alcohol consumption, urate-lowering therapy, diuretic use, proteinuria/albuminuria, inflammatory biomarkers (such as C-reactive protein and interleukin-6), and longitudinal changes in kidney function, as well as smoking status, serum lipids, and markers of CKD severity beyond eGFR (such as albuminuria category and dialysis dependence); residual confounding therefore cannot be excluded. In particular, the absence of data on urate-lowering therapy is an important limitation: because such treatment both lowers serum uric acid and is preferentially prescribed to patients with higher uric acid levels or greater comorbidity, its omission may introduce confounding by indication in either direction, and we were unable to adjust for it. Comprehensive medication data—particularly urate-lowering therapy and diuretic use—should be collected and adjusted for in future studies of this relationship. Fifth, the retrospective, single-center design limits causal inference and generalizability (including to other ethnic and geographic populations), and longitudinal variability in uric acid was not assessed. Finally, restricted cubic spline modeling was not performed and is recommended in future work to characterize potential non-linear associations. Finally, because cardiovascular events and deaths were ascertained retrospectively from routine electronic records, some outcomes may have been misclassified or incompletely captured—particularly events or deaths occurring outside the treating hospital system—and causes of death were not independently adjudicated; such outcome misclassification could bias the observed associations in either direction.

Despite these limitations, this study has important strengths: the large sample size, adjustment for significant confounders, and detailed dose–response analysis provide relevant clinical evidence. The inclusion of continuous and quartile-based analyses allowed for more precise characterization of the relationship between SUA and adverse outcomes. Furthermore, the study population reflects real-world CKD patients with substantial comorbidity burden, enhancing clinical relevance.

## 5. Conclusions

In this study, patients were diagnosed with chronic kidney disease (CKD), and hyperuricemia independently predicted mortality but was not independently associated with composite cardiac events when analyzed as a categorical variable after adjustment for traditional cardiovascular and renal risk factors. Continuous and quartile-based analyses revealed a clear dose–response relationship between SUA levels and adverse outcomes. This dose–response relationship with progressively higher uric acid concentrations is associated with a significantly high mortality risk.

This study suggests that serum uric acid behaves as a graded marker of systemic vulnerability rather than a simple binary risk factor, with progressively higher concentrations associated with higher mortality risk. These associations are consistent with the proposed pathophysiological roles of uric acid, but given the retrospective observational design and the modest-to-acceptable discriminative performance of the models, they do not by themselves justify firm recommendations for incorporating serum uric acid into routine risk-stratification frameworks. Rather, the findings indicate that serum uric acid may provide prognostic information that warrants confirmation in prospective studies with well-defined follow-up before clinical adoption.

Future longitudinal studies to analyze the mechanistic biomarkers are needed to clarify the causal role of high uric acid in cardiovascular outcomes and survival rate among patients with end-stage renal disease. Such studies should incorporate repeated (longitudinal) measurements of serum uric acid and detailed medication data, including urate-lowering therapy, to account for within-patient variability and reduce residual confounding when delineating any causal contribution of uric acid to adverse outcomes.

## Figures and Tables

**Table 1:** Baseline characteristics of patients with chronic kidney disease (*N* = 794).

Variable	Value
Age, mean ± SD (years)	68.5 ± 16.8
Male sex, *n* (%)	480 (60.5%)
Female sex, *n* (%)	314 (39.5%)
Body mass index, mean ± SD (kg/m^2^)	28.3 ± 10.0
Serum uric acid, mean ± SD (μmol/L)	401.3 ± 162.5
eGFR, mean ± SD (mL/min/1.73 m^2^)	28.7 ± 22.7
Hyperuricemia, *n* (%)	356 (44.8%)
Hypertension, *n* (%)	535 (67.4%)
Diabetes mellitus, *n* (%)	421 (53.0%)
Composite cardiac event, *n* (%)	414 (52.1%)
Death, *n* (%)	66 (8.3%)

**Table 2:** Multivariable logistic regression analysis for predictors of cardiac events (*N* = 465).

Predictor	Odds Ratio	95% Confidence Interval	*p*-Value
Hyperuricemia	1.49	0.98–2.27	0.061
Age (per year)	1.02	1.01–1.04	0.0008
Male sex	2.16	1.41–3.29	<0.001
eGFR	1.01	1.00–1.01	0.259
Diabetes mellitus	1.19	0.76–1.86	0.447
Hypertension	2.87	1.55–5.33	0.001
Body mass index	0.99	0.97–1.02	0.605

**Table 3:** Multivariable logistic regression analysis for predictors of mortality (*N* = 465).

Predictor	Odds Ratio	95% Confidence Interval	*p*-Value
Hyperuricemia	1.98	1.04–3.78	0.039
Age (per year)	1.04	1.02–1.07	<0.001
Male sex	1.18	0.61–2.28	0.618
eGFR	0.98	0.96–1.00	0.065
Diabetes mellitus	1.41	0.67–2.95	0.360
Hypertension	1.02	0.39–2.64	0.968
Body mass index	0.97	0.93–1.02	0.239

**Table 4:** Association between serum uric acid (continuous) and outcomes (*N* = 465).

Outcome	Odds Ratio per 100 μmol/L Increase	95% Confidence Interval	*p*-Value
Cardiac events	1.22	1.05–1.42	0.011
Mortality	1.29	1.09–1.53	0.004

## Data Availability

The data that support the findings of this study are available upon reasonable request from the corresponding author.

## Supplementary Materials

The following supporting information can be downloaded at: https://www.mdpi.com/article/10.3390/jcm15145479/s1, Table S1: Baseline characteristics stratified by hyperuricemia status. Values are mean ± SD or percentage; *p*-values from Student’s *t*-test or the χ^2^ test; Table S2: Baseline characteristics stratified by survival status. Values are mean ± SD or percentage; *p*-values from Student’s *t*-test or the χ^2^ test.

## References

[1] Ben Salem C., Slim R., Fathallah N., Hmouda H. (2017). Drug-induced hyperuricaemia and gout. Rheumatology.

[2] Lima W.G., Martins-Santos M.E., Chaves V.E. (2015). Uric acid as a modulator of glucose and lipid metabolism. Biochimie.

[3] Qian Y.U. (2017). Prevalence and metabolic factors of hyperuricemia in an elderly agricultural and fishing population in Taiwan. Arch. Rheumatol..

[4] Wang J., Yi J., Zhou L., Chen J., Zhang B., Huang H., Wei Y. (2025). A systematic review of the connection between serum uric acid levels and the risk of cardiovascular disease. Front. Cardiovasc. Med..

[5] Liu Y., Li Z., Xu Y., Mao H., Huang N. (2025). Uric acid and atherosclerosis in patients with chronic kidney disease: Recent progress, mechanisms, and prospect. Kidney Dis..

[6] Ponticelli C., Podestà M.A., Moroni G. (2020). Hyperuricemia as a trigger of immune response in hypertension and chronic kidney disease. Kidney Int..

[7] Filippatos G., Anker S.D., Agarwal R., Pitt B., Ruilope L.M., Rossing P., Kolkhof P., Schloemer P., Tornus I., Joseph A. (2021). Finerenone and cardiovascular outcomes in patients with chronic kidney disease and type 2 diabetes. Circulation.

[8] Hisatome I., Li P., Miake J., Taufiq F., Mahati E., Maharani N., Utami S.B., Kuwabara M., Bahrudin U., Ninomiya H. (2021). Uric acid as a risk factor for chronic kidney disease and cardiovascular disease―Japanese guideline on the Management of Asymptomatic Hyperuricemia―. Circ. J..

[9] Wang M., Zhang Y., Zhang M., Li H., Wen C., Zhao T., Xie Z., Sun J. (2021). The major cardiovascular events of febuxostat versus allopurinol in treating gout or asymptomatic hyperuricemia: A systematic review and meta-analysis. Ann. Palliat. Med..

[10] Zhao L., Cao L., Zhao T.Y., Yang X., Zhu X.X., Zou H.J., Wan W.G., Xue Y. (2020). Cardiovascular events in hyperuricemia population and a cardiovascular benefit-risk assessment of urate-lowering therapies: A systematic review and meta-analysis. Chin. Med. J..

[11] Li L., Zhao M., Wang C., Zhang S., Yun C., Chen S., Cui L., Wu S., Xue H. (2021). Early onset of hyperuricemia is associated with increased cardiovascular disease and mortality risk. Clin. Res. Cardiol..

[12] Luptáková L., Siváková D., Cvíčelová M., Wsólová L., Danková Z., Michnová A., Blažíček P. (2013). Power of biomarkers and their relative contributions to metabolic syndrome in Slovak adult women. Ann. Hum. Biol..

[13] Vorobeľová L., Falbová D., Siváková D. (2021). Differences in body composition between metabolically healthy and unhealthy midlife women with respect to obesity status. Anthropol. Rev..

[14] Feig D.I., Johnson R.J. (2003). Hyperuricemia in childhood primary hypertension. Hypertension.

[15] Nakagawa T., Tuttle K.R., Short R.A., Johnson R.J. (2005). Hypothesis: Fructose-induced hyperuricemia as a causal mechanism for the epidemic of the metabolic syndrome. Nat. Clin. Pract. Nephrol..

[16] Niskanen L.K., Laaksonen D.E., Nyyssönen K., Alfthan G., Lakka H.M., Lakka T.A., Salonen J.T. (2004). Uric acid level as a risk factor for cardiovascular and all-cause mortality in middle-aged men: A prospective cohort study. Arch. Intern. Med..

[17] Liu W.W., Yang G.B., Liu Z.Y., Guo Y., Duan L.X., Yuan J.H., Liao L., Zhang C.F., Lu J.R., Hu J. (2024). Factors influencing the occurrence of hyperuricemia and poor cardiac and renal outcomes in chronic kidney disease. Eur. Rev. Med. Pharmacol. Sci..

[18] Ndrepepa G. (2025). Uric acid and cardiovascular disease—Recent evidence on the association and underlying mechanisms. J. Lab. Precis. Med..

[19] Pai B.H., Swarnalatha G., Ram R., Dakshinamurty K.V. (2013). Allopurinol for prevention of progression of kidney disease with hyperuricemia. Indian J. Nephrol..

[20] Jalalzadeh M., Nurcheshmeh Z., Mohammadi R., Mousavinasab N., Ghadiani M.H. (2012). The effect of allopurinol on lowering blood pressure in hemodialysis patients with hyperuricemia. J. Res. Med. Sci..

[21] DeBosch B.J., Kluth O., Fujiwara H., Schürmann A., Moley K. (2014). Early-onset metabolic syndrome in mice lacking the intestinal uric acid transporter SLC2A9. Nat. Commun..

[22] Preitner F., Pimentel A., Metref S., Berthonneche C., Sarre A., Moret C., Rotman S., Centeno G., Firsov D., Thorens B. (2015). No development of hypertension in the hyperuricemic liver-Glut9 knockout mouse. Kidney Int..

[23] Gill D., Georgakis M.K., Koskeridis F., Jiang L., Feng Q., Wei W.Q., Theodoratou E., Elliott P., Denny J.C., Malik R. (2019). Use of Genetic Variants Related to Antihypertensive Drugs to Inform on Efficacy and Side Effects. Circulation.

[24] Han Y., Han K., Han X., Yin Y., Di H., Wu J., Zhang Y., Zeng X. (2022). Serum Uric Acid Might Be Positively Associated With Hypertension in Chinese Adults: An Analysis of the China Health and Nutrition Survey. Front. Med..

[25] Vargas-Morales J.M., Guevara-Cruz M., Aradillas-García C., GNoriega L., Tovar A., Alegría-Torres J.A. (2021). Polymorphisms of the genes ABCG2, SLC22A12 and XDH and their relation with hyperuricemia and hypercholesterolemia in Mexican young adults. F1000Research.

[26] Tin A., Marten J., Halperin Kuhns V.L., Li Y., Wuttke M., Kirsten H., Sieber K.B., Qiu C., Gorski M., Yu Z. (2019). Target genes, variants, tissues and transcriptional pathways influencing human serum urate levels. Nat. Genet..

[27] Song Z., Deng D., Wu H. (2024). Association of serum uric acid to all-cause and cardiovascular mortality in patients with cardiovascular disease. Sci. Rep..

[28] Liu W.C., Hung C.C., Chen S.C., Yeh S.M., Lin M.Y., Chiu Y.W., Kuo M.C., Chang J.M., Hwang S.J., Chen H.C. (2012). Association of hyperuricemia with renal outcomes, cardiovascular disease, and mortality. Clin. J. Am. Soc. Nephrol..

[29] Vorobeľová L., Danková Z., Candráková-Čerňanová V., Falbová D., Cvíčelová M., Beňuš R., Siváková D. (2019). Association of the ESR1 polymorphism with menopause and MLXIPL genetic variant influence serum uric acid levels in Slovak midlife women. Menopause.

[30] Mufti N.U., Khan R.M., Haseeb A.B., Sardar W., Hanan S., Khan S.A. (2022). Hyperuricemia in patients with chronic renal failure: A single center study, Pakistan. Pak. J. Med. Health Sci..

[31] Kwon Y.E., Ahn S.Y., Ko G.J., Kwon Y.J., Kim J.E. (2024). Impact of Uric Acid Levels on Mortality and Cardiovascular Outcomes in Relation to Kidney Function. J. Clin. Med..

[32] Xu X., Huang J., Wu S., Ji Q., Guo X., Huang Y. (2021). The Association between the Serum Uric Acid Level and Hypertension in Middle-Aged and Elderly Adults. Cardiovasc. Ther..

