# Supplementary material for: Serum Uric Acid and Mortality Risk in Chronic Kidney Disease: A Dose–Response Analysis
Source: J Clin Med. 2026 Jul 13;15(14):5479. doi: 10.3390/jcm15145479 (PMC13413096; doi:10.3390/jcm15145479)
Supplement: Supplementary file 1 [file jcm-15-05479-s001.zip › jcm-4405465-supplementary.pdf]

Table S1. Baseline characteristics stratified by hyperuricemia status. Values are mean ± SD or percentage; p-values from Student's t-test or the χ^2 test.

Variable	Non-hyperuricemic (n = 431)	Hyperuricemic (n = 359)	p
Age, years	67.9 ± 17.2	69.1 ± 16.4	0.300
Male, %	64.7	55.7	0.012
BMI, kg/m^2	27.4 ± 8.0	30.0 ± 14.9	0.005
Serum uric acid, µmol/L	292 ± 79	532 ± 137	<0.001
eGFR, mL/min/1.73 m^2	31.5 ± 25.2	25.5 ± 18.9	<0.001
Hypertension, %	64.7	69.9	0.142
Diabetes, %	51.0	52.6	0.706
Smoking, %	9.7	6.7	0.156
Total cholesterol, mmol/L	3.59 ± 1.15	3.89 ± 1.44	0.003
LDL, mmol/L	1.94 ± 0.96	2.17 ± 1.08	0.004
HDL, mmol/L	1.19 ± 0.39	1.16 ± 0.41	0.456
Triglycerides, mmol/L	1.27 ± 0.76	1.43 ± 0.91	0.014
Urate-lowering therapy, %	4.2	7.8	0.044
Diuretic use, %	28.3	39.0	0.002
Cardiac events, %	49.2	54.0	0.198
Mortality, %	5.1	12.3	<0.001

Table S2. Baseline characteristics stratified by survival status. Values are mean ± SD or percentage; p-values from Student's t-test or the χ^2 test.

Variable	Survivors (n = 729)	Non-survivors (n = 66)	p
Age, years	67.6 ± 16.8	76.9 ± 14.3	<0.001
Male, %	60.6	60.6	1.000
BMI, kg/m^2	28.8 ± 12.0	26.1 ± 6.4	0.007
Serum uric acid, µmol/L	391 ± 151	508 ± 228	<0.001
eGFR, mL/min/1.73 m^2	29.2 ± 23.0	23.3 ± 19.7	0.039
Hypertension, %	64.5	92.4	<0.001
Diabetes, %	49.7	72.7	<0.001
Smoking, %	8.2	9.1	0.992
Total cholesterol, mmol/L	3.76 ± 1.29	3.37 ± 1.31	0.031
LDL, mmol/L	2.06 ± 1.02	1.87 ± 1.03	0.192
HDL, mmol/L	1.18 ± 0.40	1.06 ± 0.45	0.041
Triglycerides, mmol/L	1.36 ± 0.85	1.18 ± 0.69	0.064
Urate-lowering therapy, %	5.1	13.6	0.010
Diuretic use, %	30.3	62.1	<0.001
Cardiac events, %	47.6	90.9	<0.001
